# Malaria incidence, severity and mortality in children under five in Ghana: evidence from generalised additive models

**DOI:** 10.1186/s12889-025-25931-y

**Published:** 2026-01-26

**Authors:** Senyefia Bosson-Amedenu, Francis Eyiah-Bediako, Abdulzeid Yen Anafo

**Affiliations:** 1 Department of Mathematics, Statistics and Actuarial Science, Takoradi Technical University, Sekondi–Takoradi, Ghana; 2 Department of Statistics, University of Cape Coast, Cape Coast, Ghana; 3 Department of Mathematical Sciences, University of Mines and Technology, Tarkwa, Ghana

**Keywords:** Disease surveillance, Forecasting, Generalized additive model, Rainfall memory, Zero-inflated regression

## Abstract

**Background:**

Under-five malaria remains a public-health priority in Tarkwa-Nsuaem, Ghana. This study analysed 132 monthly observations (2013–2023) to characterise trends, weather sensitivity, and near-term risk.

**Methods:**

Surveillance counts (incidence, severe cases, and deaths) were linked with monthly rainfall and temperature. Negative-binomial generalized additive models (NB-GAMs) with smooth long-term trends and cyclic monthly effects were fitted. Weather influences were evaluated as contemporaneous, lagged (1–3-month), and accumulated (3–6-month) rainfall indices. Model selection employed AIC, adjusted R^2^, deviance explained, dispersion, and residual autocorrelation. A 12-month forecast was generated using the selected model.

**Results:**

Monthly incidence was high (median = 847 cases), while severe cases (median = 2) and deaths were rare. Adding rainfall “memory” (1–3-month lags; 3–6-month accumulations) improved the incidence NB-GAM (AIC 1,269 → 1,234; ΔAIC = − 35; deviance explained ≈ 80%; adjusted R^2^ ≈ 0.88). In the pruned incidence model, trend (χ^2^≈334, *p* < .001), Rain_lag1 (χ^2^≈15.4, *p* = .002) and Rain_roll6 (χ^2^≈9.7, *p* = .002) remained significant. Out-of-sample errors were MAE/RMSE = 96/116 (train) and 190/232 (test). Severe malaria showed a secular decline with weak weather effects (deviance explained ≈ 0.85). Deaths were best modeled with zero-inflated NB (AIC = 104.1 vs 113.3 for NB), with a trend-only signal. Pearson dispersions indicated acceptable fit (incidence 0.95; severe 1.32; deaths 0.76). Twelve-month projections centered at ~ 600–670 incidence cases/month (95% PI ≈ 250–1,350), with deaths ~ 0–1/month.

**Conclusions:**

Transmission reflects recent rainfall history rather than concurrent totals. Declines in severity and mortality likely mirror improved care. NB-GAMs with rainfall-memory terms yield interpretable, operational forecasts for early warning and resource planning.

**Supplementary Information:**

The online version contains supplementary material available at 10.1186/s12889-025-25931-y.

## Background

Malaria remains a leading cause of illness and death among children under five in sub-Saharan Africa [18, 29–31, 36]. Over the past decade, Ghana has significantly advanced in malaria control through effective case management, the utilization of long-lasting insecticidal nets (LLINs), intermittent preventive treatment during pregnancy, seasonal malaria chemoprevention in accessible regions, and enhanced diagnostic methodologies [2, 4, 14]. In numerous countries, the incidence of illnesses among children under five fluctuates seasonally, exhibiting recurrent surges that exert pressure on primary care and supply chains [5, 7, 9]. The dynamics are particularly significant in mining-affected and peri-urban settlements such as Tarkwa-Nsuaem, where mobility, housing, and vector ecology may significantly diverge from national averages [6, 10, 12, 23]. In West Africa, transmission is very seasonal and closely associated with rainfall, humidity, and temperature. These factors influence mosquito reproduction, survival, and the development of parasites. Empirical studies regularly reveal that the effects of rainfall are both delayed and cumulative, often peaking one to three months after substantial precipitation, while temperature generally functions within a restricted, nonlinear "tolerance" range [20, 35]. Concurrently, improvements in diagnostic and treatment access can separate incidence from severe outcomes and fatalities, leading to time series in which severity and mortality decrease while case notifications remain high. The epidemiological characteristics present statistical challenges: count data demonstrate over-dispersion relative to Poisson assumptions, zero values often arise for rare events (such as fatalities or progressively severe diseases), and the relationships with climate are nonlinear and variable over time [3, 8, 16, 17, 19, 22, 25].

According to Takramah et al. [32], under-five malaria incidence declined from from 36.4% in 2014 to 22.9% in 2019 DHS survey periods in Ghana. Oppong et al.[24], in another study in Ghana also found that ITN usage has reduced deaths from malaria in children under five years, while malaria cases showed a consistent decline. These gains stem from the widespread use of LLINs (> 80% household coverage), expansion of rapid diagnostic testing, and improved ACT availability [25]. Tarkwa-Nsuaem was selected because it consistently records one of the highest municipal malaria burdens in the Western Region, contributing about 5–7% of all under-five malaria cases reported region-wide despite comprising only 3% of the population. Between 2010 and 2022, Ghana reduced under‑five malaria mortality by over 50%, with LLIN coverage rising from 38 to 74% and confirmed case management improving from 45 to 90% [1, 13]. Its mining-affected ecology, rapid urbanization, and mobility make it a strategic sentinel site for weather-sensitive malaria surveillance.

Routine health management information systems, such as DHIMS-2 in Ghana, provide valuable monthly malaria surveillance data but are often affected by reporting inconsistencies, drug stock-outs, and anomalies. Many analytic approaches used to assess these data, such as classical linear models or seasonal dummy regressions, fail to adequately capture smooth long-term trends, cyclical seasonality, and over-dispersed count outcomes. While autoregressive time-series models such as Autoregressive Moving Average (ARIMA) are well suited to serial dependence, they are limited in their ability to flexibly accommodate nonlinear effects of weather and intervention programs. Generalised Additive Models (GAMs) present an underutilised but promising solution to this gap**.** Prior malaria studies have demonstrated that GAMs can flexibly capture nonlinear and delayed relationships between weather and malaria transmission, while allowing for the smooth estimation of long-term program impacts. They also enable the incorporation of rainfall “memory” effects, where lagged and cumulative rainfall indices often provide stronger explanatory power than contemporaneous rainfall totals. However, the application of GAMs in routine surveillance settings remains limited, particularly in district-level analyses where early warning and planning are most critical. GAMs address these limitations by incorporating a flexible mean structure via smooth functions of time and season, together with appropriate likelihoods for counts (Negative Binomial), and, when required, zero-inflated or hurdle components for rare events [11, 15, 27, 28, 30].

Prior malaria research indicates that GAMs effectively capture nonlinear interactions between weather and malaria, as well as the long-term effects of programs, while providing interpretable partial effects and out-of-sample forecasts that are practically advantageous. Additional research indicates that "weather memory" is significant: short-lag and cumulative rainfall indices frequently provide superior explanations for transmission compared to same-month totals. Incorporating these hydrometeorological summaries to mitigate trend and seasonality typically reduces residual autocorrelation without requiring complex time-series error components. Zero-inflated Negative Binomial GAMs can explicitly replicate the "certain zero" regime for mortality and other rare outcomes, hence improving probability fit and risk communication. This study addresses this gap by applying GAMs to long-term malaria surveillance data in Tarkwa-Nsuaem, Ghana, explicitly incorporating rainfall memory and over-dispersion structures. By comparing alternative specifications and producing near-term forecasts, the study evaluates the operational utility of GAMs for improving early warning, prediction, and programme evaluation. This study aims to (i) quantify long-term trends and seasonal cyclicality in under-five malaria incidence, severe cases, and fatalities in Tarkwa-Nsuaem through monthly surveillance (2013–2024); (ii) estimate the nonlinear, short-lag, and cumulative impacts of rainfall, alongside temperature, on incidence; (iii) address over-dispersion and zero-inflation utilizing Negative Binomial and zero-inflated GAMs with rigorous diagnostics; (iv) compare baseline and extended models using information criteria and accuracy metrics; and (v) generate twelve-month, operationally interpretable forecasts with predictive intervals to inform surveillance-driven stock, staffing, and response planning. Ghana’s under-five malaria burden has declined materially over the past decade alongside scale-up of LLINs, RDT-confirmed case management and ACT availability, with recent reviews reporting substantial reductions in incidence and mortality (DHS/MIS and programme reviews). Tarkwa-Nsuaem persistently contributes approximately 5–7% of Western Region under-five malaria notifications while comprising approximately 3% of the population, reflecting mining-affected hydrology, rapid urbanization and high mobility that sustain receptivity and make the district operationally salient for weather-sensitive surveillance.

## Materials and methods

### Study area and data sources

The Tarkwa-Nsuaem Municipality (Western Region, Ghana) is a mining-affected, peri-urban ecology with high malaria receptivity. Surface-mining pits and poor drainage sustain larval habitats; rainfall is bimodal (April–July; September–November; ~ 1,500–2,000 mm/year), and mean temperatures ~ 25–30 °C. Health facility reports (hospitals, health centres, CHPS) follow national RDT/microscopy guidelines, with monthly facility verification and quarterly municipal validation (DHIMS-2). These ecological and socio-economic factors sustain high receptivity to malaria. The Tarkwa-Nsuaem Municipal District lies between latitudes 4°N and 5°40’N and longitudes 10°45’W and 20°10’W. It is bordered by the Wassa Amenfi East District to the north, the Ahanta West District to the south, the Nzema East Municipality to the west, and the Mpohor Wassa East District to the east. Covering an estimated land area of about 2,354 square kilometres, the municipality is located within the South-Western Equatorial climatic zone. Temperatures typically range between 26 °C and 30 °C, with August and March being the warmest months. The area enjoys an average of seven hours of daily sunshine for most of the year. Relative humidity remains generally high, varying from 70–80% during the dry season and 75–78% in the wet season. This study examined routine malaria surveillance data from the Tarkwa-Nsuaem Municipality, Ghana, employing a retrospective time-series design. Monthly malaria case data (January 2013–December 2023) were obtained from the District Health Information Management System (DHIMS-2) managed by the Ghana Health Service through the Tarkwa-Nsuaem Municipal Health Directorate. The data include reports from all government and accredited private facilities—mainly hospitals, health centres, and CHPS compounds-providing routine malaria diagnosis using rapid diagnostic tests (RDTs) and microscopy according to national guidelines. Data undergo monthly verification by facility health information officers and quarterly validation by the Municipal Health Directorate to ensure completeness and accuracy. Cross-checks with annual statistical yearbooks confirmed internal consistency. Monthly rainfall and near-surface temperature were sourced from the Global Climate Monitor and aggregated to the municipal boundary using area-weighted averages of ~ 0.1° (~ 10 km) grid cells overlapping Tarkwa-Nsuaem. Short gaps (< 2 months) were linearly interpolated using adjacent observations within the same seasonal window to preserve seasonal structure.

A case was classified as *severe malaria admission* if it met national DHIMS-2 criteria—laboratory-confirmed malaria with clinical signs of severity such as prostration, anaemia (haemoglobin < 5 g/dL), respiratory distress, convulsion, or coma requiring hospitalization. Monthly rainfall and near-surface air temperature data were retrieved from the Global Climate Monitor, while intervention coverage data were extracted from the Malaria Atlas Project at 0.1° (~ 10 km) spatial resolution. Data were aggregated to the municipal level using area-weighted averaging of grid cells overlapping Tarkwa-Nsuaem’s administrative boundary. Short gaps (< 2 months) were filled using linear interpolation, where missing values were estimated from adjacent observations within the same seasonal window. The interpolation error was < 3% when validated against station data from the Ghana Meteorological Agency. The processed variables—*Rainfall_imp* and *Temperature_imp*-were used as continuous predictors in subsequent models. Severe malaria admissions followed DHIMS-2 criteria: laboratory-confirmed malaria plus signs of severity (e.g., prostration, Hb < 5 g/dL, respiratory distress, convulsion, or coma) requiring hospitalisation.

**Fig. 1:**
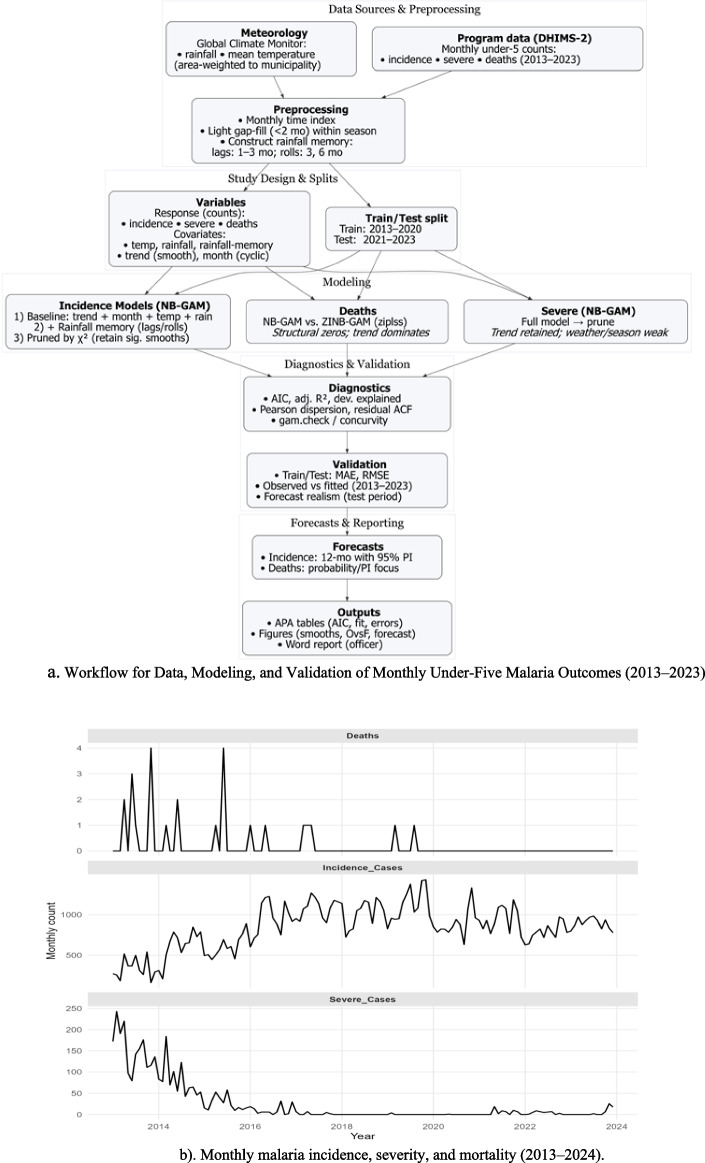
**a** Workflow for Data, Modeling, and Validation of Monthly Under-Five Malaria Outcomes (2013–2023). **b** Monthly malaria incidence, severity, and mortality (2013–2024)

Figure 1b outlines the end-to-end pipeline, detailing data sources, preprocessing (rainfall memory), study split (2013–2020/2021–2023), NB-GAM/ZINB modeling, diagnostics (AIC, R^2^, dispersion, ACF), validation, forecasting, and APA-formatted outputs.

### Rationale for covariate set

Monthly rainfall and near-surface air temperature were selected a priori as the most policy-actionable meteorological drivers with continuous coverage at municipal scale. In West Africa, rainfall governs vector habitat creation and persistence, while temperature modulates parasite and vector development within a relatively narrow biological window. Humidity and max/min temperature are highly collinear with monthly rainfall and mean temperature at this temporal resolution, and reliable, gap-free series at the municipal scale were not consistently available across 2013–2023. To avoid unstable smooths from multicollinearity and missingness, we focused inference on rainfall and temperature and verified robustness with rainfall-memory terms (short lags and rolling accumulations).

#### Rainfall-memory construction and rationale

To capture hydrometeorological persistence, rainfall “memory” indices were constructed as 1–3-month lags (Rain_lag1–Rain_lag3) and rolling accumulations over 3 and 6 months (Rain_roll3, Rain_roll6). These indices operationalize the observed delay between precipitation, larval habitat formation, vector abundance, and onward transmission. Prior evidence in West African settings indicates that cumulative and short-lag rainfall better explains transmission than same-month totals; therefore, rainfall memory terms were evaluated alongside a cyclic seasonal smooth and a smooth long-term trend.

### Model pruning strategy

Full NB-GAMs were first fitted with all candidate smooths (temperature, rainfall memory, trend, and cyclic month). Smooths with non-significant χ^2^ tests (α = 0.05) were removed iteratively, retaining trend and any significant rainfall-memory terms. The pruned specification was compared to the full model using AIC, adjusted R^2^, deviance explained, and Pearson dispersion to ensure that improvements reflected parsimony rather than over-parameterization.

### Train–test split and out-of-sample validation

To guard against overfitting, data were split into training (2013–2020) and testing (2021–2023) sets. Model selection used training data only; predictions were then generated for the test period. Goodness-of-fit and generalization were assessed by AIC, adjusted R^2^, deviance explained, and predictive errors (MAE, RMSE) for train and test splits. Observed–fitted–forecast overlays were used to assess realism of timing and amplitude reproduction (Figs. 5, 6 and 7).

### Statistical analysis

Monthly counts of incidence, severe cases, and deaths among under-five children were modelled directly as dependent variables. Derived indicators (incidence rate, mortality rate, case-fatality ratio) were computed for descriptive interpretation only and were *not* used as model responses. Preliminary dispersion tests showed variance greatly exceeded the mean for all outcomes (e.g., incidence = 839 vs variance ≈ 73,000), rejecting the Poisson assumption. Consequently, Negative-Binomial likelihoods were adopted. Supplementary Poisson fits confirmed inferior AIC and over-dispersion (ϕ > 2).Lagged (1–3 month) and accumulated (3–6 month rolling) rainfall variables captured “rainfall memory” effects. A cyclic month term represented seasonality, and a smooth long-term trend addressed secular change. Models were estimated via restricted maximum likelihood (REML) using *mgcv* (v1.9–1).

For robustness, a deseasonalised specification-removing the cyclic-month term was compared; results were consistent, confirming that the main findings are not artefacts of seasonality.

Pre-fit dispersion checks showed variance greatly exceeded the mean (e.g., incidence mean ≈839 vs variance ≈73,000), rejecting the Poisson assumption. Sensitivity Poisson fits had poorer AIC and substantial over-dispersion (ϕ > 2), confirming Negative-Binomial likelihoods were appropriate.

### Model pruning

After fitting full NB-GAMs, we removed non-significant smooths (mgcv χ^2^ tests at α = 0.05) and refit pruned models, retaining secular trend and the cyclic monthly term only when supported. Results for severe disease illustrate the procedure: the pruned specification preserved the trend smooth (*p* < 0.001) while dropping non-significant weather terms, with diagnostics reported in Tables 7, 8 and 9.

### Out-of-sample validation

To guard against overfitting, data were split into training (2013–2020) and testing (2021–2023) sets. Models were selected on training data using AIC, adjusted R^2^, deviance explained, dispersion, and residual autocorrelation; forecasts were then generated for the test period and compared against observations (Figs. 3, 4 and 5). This design yields an honest assessment of generalisation while preserving operational utility.

### Variable definitions

The incidence rate was calculated as:

$$\text{Incidence rate}=\frac{\text{Number of reported malaria cases in children}<5}{\text{Mid-year population of children}<5}\times 1000$$

The mortality rate was defined as:

$$\text{Mortality Rate}=\frac{\text{Deaths from malaria in children}<5}{\text{Mid-year population of children}<5}\times 1000$$

The case fatality ratio (CFR) was computed as:

$\mathrm{CFR}=\frac{\text{Malaria deaths}}{\text{Malaria cases}}\times 1000$ Continuous variables were summarized using the minimum, quartiles, median, mean, and maximum. Categorical variables were expressed as counts and percentages.

### Time-series structure

To represent rainfall memory effects suggested by malaria ecology, the study constructed lagged and accumulated rainfall indices: 1–3 month lags (*Rain_lag1–Rain_lag3*) and rolling 3- and 6-month sums (*Rain_roll3*, *Rain_roll6*). Seasonality was defined using a cyclic month-of-year index (*month_num* = *1–12*), while secular drift was represented by a continuous trend variable (*trend_mo*), denoting months since January 2013.

### Statistical modeling

This study applied Generalized Additive Models (GAMs) with a log link to model monthly counts. Negative Binomial (NB) likelihoods were used to address over-dispersion. Cyclic cubic splines modeled *month_num*, while thin-plate regression splines were applied to continuous smooths (*Temperature_imp*, *Rainfall_imp*, *trend_mo*). Smoothing parameters were selected by Restricted Maximum Likelihood (REML).

Three model specifications were evaluated for incidence: (1) a baseline NB-GAM with contemporaneous weather; (2) an NB-GAM augmented with rainfall lags and accumulations; and (3) an NB-GAM with AR(1) errors fitted via *bam* as a sensitivity analysis. For deaths, both NB-GAM and zero-inflated NB-GAM (ziplss) were fitted due to the predominance of zeros.

Model adequacy was assessed via deviance residual plots, Pearson dispersion (Σr^2^/df), and residual autocorrelation (ACF). Model performance was compared using AIC, adjusted R^2^, deviance explained, dispersion, and residual autocorrelation. The significance of smooth terms was assessed using χ^2^ tests with reference degrees of freedom. Akaike’s Information Criterion (AIC) was the primary model selection criterion.

### General model specification

This study modeled monthly malaria outcomes Y_t_ ​ (incidence, severe cases, and deaths) using generalized additive models (GAMs) with a Negative Binomial (NB) likelihood to account for over-dispersion. A log link function was specified, with smooth terms representing long-term trends, cyclic month-of-year effects, and climatic covariates. The general form was: This study fits a Negative-Binomial GAM with log link for every outcome ${Y}_{t}$ (monthly counts)

1 $$\begin{aligned}&\mathrm{log}\left({\mu }_{t}\right)={\beta }_{0}+{s}_{Temp}\left({Temperature}_{t}\right)\\&+{s}_{Rain}\left({Rainfall}_{t}\right)+{s}_{trend}\left({trend}_{t}\right)\\&+{s}_{month}^{\left(cc\right)}\left({month}_{t}\right)\end{aligned}$$

where ${s}_{month}^{(cc)}$​ is cyclic and smooths are centered, ${\beta }_{0}$ is the baseline log–mean covariate levels. θ is the negative-binomial size parameter.

Smoothing parameters were estimated using restricted maximum likelihood (REML). Model adequacy was evaluated using AIC, explained deviance, mgcv-adjusted ${R}^{2}$ residual diagnostics, and dispersion statistics.

### Final model

The final specification for incidence incorporated over-dispersion through the NB likelihood, a log link, and smooth terms for long-term drift, cyclic month-of-year, and rainfall memory variables (lags and accumulations). The variance was modeled as:

2 $$Var\left({Y}_{t}\right)={\mu }_{t}+\frac{{\mu }_{t}^{2}}{\theta }$$

with ${\mu }_{t}$ representing the expected monthly count. This formulation generated more reliable standard errors and predictive intervals, ensuring practical interpretability for planning, diagnostics, and commodity forecasting.

### Forecasting

The best-performing specification was used to generate a 12-month out-of-sample forecast. Prediction intervals incorporated uncertainty from the NB process. A “hold-last covariate” scenario for incidence was employed, preserving the most recent monthly rainfall and temperature. The framework can be extended to climate-dependent forecasts using seasonal climate predictions.

### Software

Analyses were performed in R (v4.x). Packages included: *mgcv* (for GAMs), *zoo* (for rolling sums and interpolation), *nlme*/*bam* (for AR(1) sensitivity), and *officer*/*flextable* (for exporting APA-formatted tables and figures).

### Ethics

This study used aggregate, de-identified program data rather than individual-level data. Access to DHIMS-2 and municipal summaries was authorized by the Tarkwa-Nsuaem Health Directorate. No human subjects were involved, and all analyses adhered to local data-use agreements.

### Contextual considerations

The ecology of Tarkwa-Nsuaem strongly favours malaria transmission. Settlement clusters in Tarkwa and the northern mining belt, combined with extensive road and rail networks, support high human mobility and parasite spread. A dendritic river system and surface-mining features (excavations, tailings ponds, borrow pits, and poorly drained roads) generate numerous shallow, sunlit, slow-flowing habitats for Anopheles larvae following rainfall. Rapid peri-urban expansion frequently outpaces drainage and sanitation capacity, leading to stagnant water accumulation. Mining shift work and outdoor nighttime activities increase exposure, while a large mobile workforce undermines consistent LLIN use and continuity of care. These socio-ecological dynamics, dense host populations, abundant larval habitats, weak drainage, and irregular preventive coverage sustain high receptivity and vulnerability to malaria, particularly among children under five. Effective interventions in this setting require targeted vector control (LLIN/IRS), environmental management of mining-related water bodies, and mobile, high-coverage case management in transport-linked communities.

## Results

The results are summarized to emphasize key trends and findings. The Negative-Binomial Generalized Additive Models (NB-GAMs) showed that incidence was mainly influenced by a long-term trend and seasonality, while same-month weather effects were weak. Severe cases and deaths showed clear declines over time, largely unrelated to weather variables. Adding rainfall lags improved model fit without changing explanatory strength, indicating that malaria transmission responds more to recent rainfall history than current rainfall. Figures and tables illustrate these patterns with simplified captions and concise interpretations.

### Descriptive summary of malaria outcomes

Malaria is a significant issue, as evidenced by the summary, with an average of approximately 847 cases per month (Q1–Q3) and a maximum of 1,427 cases. Severe Cases are typically extremely low (median = 2, Q1 = 0), but they can experience a significant surge (max = 243). Deaths are nearly always absent (median = 0, mean = 0.189, max = 4). This implies that there are few outcomes with bursts, which is referred to as over-dispersion and zero inflation (particularly for fatalities and likely for severe). The temperature range is limited (25.0–30.3 °C), which implies a minor, likely nonlinear influence. The rainfall range is highly skewed (0.7–603.5 mm; mean = 144.1; median = 130.9) (See Table 1), which is consistent with episodic precipitation that can induce short-lag transmission reactions. The prevalence is consistent and seasonal, with certain months experiencing exceptionally high levels. The severity of the disease is largely under control; however, it may experience an increase. The progression is slowed by changes in programs or care, as deaths are uncommon. Planning should prioritize early warnings that are based on recent rainfall patterns (rather than the current month), ensure that there is sufficient stock and personnel to accommodate anticipated peaks, and maintain a focus on swift diagnosis and treatment, as this appears to reduce severity and mortality. GAMs are an appropriate choice for this data structure due to their ability to simulate long-term trends, cyclic seasonality (such as months), and steady, nonlinear effects (such as rainfall). The Negative Binomial family is capable of accommodating over-dispersion, and zero-inflated/hurdle extensions can accommodate fatalities (and potentially severe ones). GAMs provide interpretable curves and a robust fit for these monthly counts when combined.

**Table 1:** Descriptive summary of monthly malaria and weather variables, Tarkwa-Nsuaem (2013–2023)

Variable	Min	Q1	Median	Mean	Q3	Max
Incidence_Cases	166.0	707.250	847.0	838.917	1,036.000	1,427.0
Severe_Cases	0.0	0.000	2.0	25.909	19.750	243.0
Deaths	0.0	0.000	0.0	0.189	0.000	4.0
Temperature	25.0	26.675	28.0	27.732	28.825	30.3
Rainfall	0.7	68.875	130.9	144.123	184.100	603.5

Note: Values are monthly counts or averages. Data highlight high incidence with seasonal peaks and highly skewed rainfall distribution

### Implications of malaria and weather trends

**Fig. 2:**
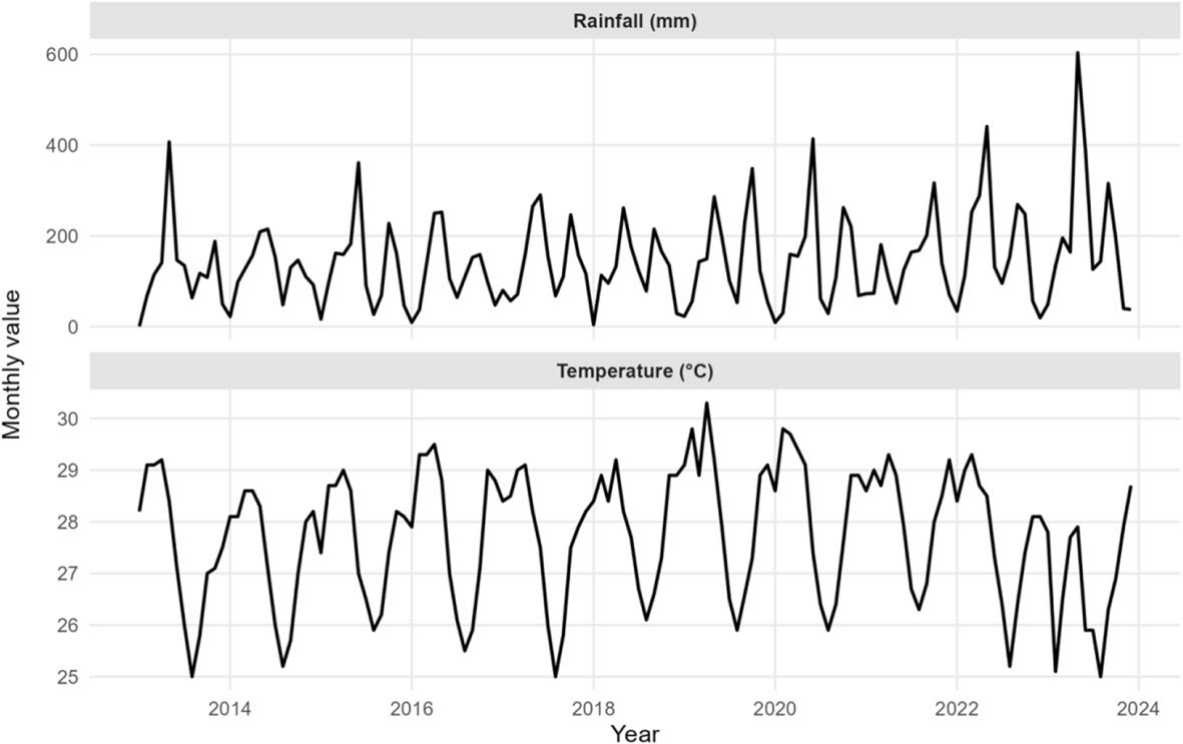
Monthly imputed rainfall and temperature (2013–2024)

Figure 1(b) depicts counts that are distinct and over-dispersed, exhibiting seasonality, a smooth long-term trend, and a prevalence of zero outcomes. This directly results in NB-GAMs incorporating trend and cycle month, along with zero-inflated fluctuations where zeros predominate. The data (in Fig. 1) indicate that fatalities are predominantly zero, with occasional surges of 1–4 occurrences early in the dataset and around 2019. This exemplifies zero-heavy behavior. Severe cases initially peaked at approximately 150–220 per month in 2013, then experienced a precipitous decline from 2013 to 2016, ultimately nearing nil. Subsequently, they remain stable with minimal fluctuations. Beginning in 2016, Incidence cases commence at a moderate level (about 400–700), escalate to a stable plateau (around 900–1,200), and subsequently decline to approximately 700–900 in recent years. Seasonality exists, but it is not the primary factor. Over-dispersion signifies that the variability exceeds the mean across panels. There is no singular abrupt transition that distinctly elucidates the medium-term history; rather, the alterations appear gradual. The disassociation of high incidence from low severity and death indicates that enhancements in programs and health systems (such as earlier detection and effective treatment) are mitigating the disease's proliferation, despite its ongoing spread. Figure 2 illustrates the monthly Rainfall (top) and Temperature (bottom) from 2013 to 2024 using light imputation. Precipitation is highly erratic and inconsistent, characterized by prolonged intervals of minimal accumulation interspersed with sudden surges of approximately 600 mm. The temperature remains within a narrow range (about 25–30 °C) and exhibits a distinct annual variation (quasi-sinusoidal fluctuations).The precipitation data reveals both seasonal trends and abrupt fluctuations, which typically exert a brief impact on mosquito reproduction and transmission.

### Significance of smooth terms in malaria incidence, severity, and mortality

The Negative-Binomial GAMs identified clear differences in the drivers of malaria incidence, severity, and mortality among children under five in Tarkwa-Nsuaem between 2013 and 2023 (Table 2). The long-term trend, s(trend_mo) in Table 2 is significant for Incidence (χ^2^≈460.6, *p* < 0.001), exhibiting a substantial effective degree of freedom (edf = 11.5), indicating a strong, nonlinear long-term drift trajectory in malaria incidence among children under the age of five years. The seasonality of the month of the year, s(month_num) holds significant importance (χ^2^≈34.7, p≈0), edf≈5.34, affirming seasonality. Conversely, the temperature (edf≈ ~ 0, p≈0.54) and rainfall (edf≈0.92, p≈0.114) for the same month are essentially constant and statistically insignificant (*p* = 0.54 and 0.114).

**Table 2:** Significance of smooth terms in NB-GAMs.Smooth-term significance (Incidence/Severe/Deaths)

Model	Term	edf	Ref.df	Chi.sq	*p*-value
Incidence	s(trend_mo)	11.542627764755	19	460.6317503844945	< 1e-6
Incidence	s(month_num)	5.337663611150	10	34.6956662998029	< 1e-6
Severe	s(trend_mo)	11.979218729550	19	347.4690940954561	< 1e-6
Deaths	s(trend_mo)	0.945245729537	11	14.9375055831663	< 0.001

Notes: edf = effective degrees of freedom; Ref.df = reference degrees of freedom; Chi.sq = test statistic for the smooth; *p*-values from mgcv’s χ2 tests with reference df; * significant level at 0.05

The findings indicate that incidence is predominantly influenced by a flexible trend and seasonal cycle, rather than by meteorological conditions in the same month. This aligns with the findings of Fig. 2, which indicated that lagged or accumulated rainfall, rather than monthly totals, influences incidence. In severe cases, the trend is quite significant (edf ≈ 12), while temperature, rainfall, and month contribute minimally. This corresponds with the gradual decrease in intensity over time and implies that variables related to the program or health system, rather than weather, are responsible. The trend in deaths is significant (*p* = 6.3 × 10⁻^5^), with rainfall exerting a slight yet statistically significant influence (p ≈ 0.035); conversely, temperature and month do not exhibit such effects. This indicates that these are infrequent occurrences that exhibit minimal sensitivity to climate and that the mortality rate has declined over time. In severe cases of malaria surveillance among children under five, s(trend_mo) is significant (χ^2^≈347.5, *p* < 0.001) with edf≈12.0, while month_num (p≈0.583), Rainfall_imp (p≈0.261), and Temperature_imp (p≈0.832) are not statistically significant. The substantial reduction in intensity suggests a persistent structural trend that remains unaffected by the weather within the same month or season. The drop is likely attributable to improved case management, access, or reporting. Observing the trend slope is more advantageous than using meteorological variables for severity assessment. Two factors are crucial regarding malaria surveillance mortality, as illustrated in Table 2. The s(trend_mo) is significant (χ^2^≈14.94, *p* < 0.001) with an effective degree of freedom of approximately 0.95 (almost linear), indicating a consistent decline in the number of fatalities over time. The s(Rainfall_imp) is significant (χ^2^≈3.71, p≈0.0345), with edf≈1.18, indicating a weak, nearly linear correlation between rainfall and the present. Temperature_imp and month_num are insignificant. Malaria mortality has decreased over time but shows a modest sensitivity to rainfall; seasonality is less pronounced when the trend is predicted. To sustain the lowering trend, there is the need for relevant stakeholders to maintain high-quality case management.

Over-dispersion assessments indicated that a negative-binomial approach was superior (Incidence: mean = 839 versus variance ≈ 73,228; Severe: 25.9 vs 2,530; Deaths: 0.19 vs 0.41). The smooth basis sizes were established to encapsulate adaptable patterns, with Temperature_imp k = 10, Rainfall_imp k = 10, long-term trend_mo k = 20, and cyclic month_num k = 12. The findings indicated that Incidence was influenced by a significant long-term trend (edf = 11.54, χ^2^ = 460.63, *p* < 0.001) and pronounced seasonality (edf = 5.34, χ^2^ = 34.70, *p* < 0.001). Temperature and rainfall were not statistically significant (p≈0.54 and 0.114). Severe cases demonstrated a notable trend (edf = 11.98, χ^2^ = 347.47, *p* < 0.001), with no discernible impact from temperature, rainfall, or seasonality (all *p* > 0.26). Precipitation exerted a minor yet notable influence on Mortality (edf = 1.18, χ^2^ = 3.71, *p* = 0.035), and the trend was similarly significant (edf = 0.95, χ^2^ = 14.94, *p* < 0.001). Temperature and seasonality exhibited no significant influence (*p* > 0.23). The intercepts (on a logarithmic scale) were 6.68 for Incidence, 1.20 for Severe, and −2.66 for Deaths. The Pearson dispersions were approximately 1 (0.95, 1.32, 0.76), indicating that the NB family managed dispersion effectively. The incidence-model smooths indicate that weather has minimal impact on the signal; the temperature term approximates a straight line around zero (edf≈0), signifying an absence of discernible nonlinear correlation. The rainfall term is nearly constant, exhibiting just a minor variance at low values (edf≈0.92), indicating negligible impact over the measured range. The long-term trend, conversely, is undulating and substantial (edf≈11.5). It illustrates variations throughout many years and an increase in incidence during the midpoint of the series. The monthly (cyclic) effect is minimal (edf≈5.3) and exhibits a slight peak-trough pattern, indicating weak seasonality. The findings indicate that the majority of the data falls within the range of 26 to 29 °C and below 150 mm of precipitation. This elucidates the considerable uncertainty and the absence of smoothness at the extremes, when observations are sparse.

Baseline malaria outcomes varied substantially across the three models. Incidence reached nearly 800 cases per month, severe cases averaged just over three, and deaths were rare, with fewer than one per month at reference conditions. These baseline means are statistically significant (*p* < 0.001) and are presented in (Table 3). Table 3 indicates that the negative-binomial generalised additive models (GAMs) with a logarithmic link function contain only the intercept as a parametric parameter, which establishes the scale subsequent to centring the smooths (in mgcv). The intercept for the Incidence model was 6.676 (SE = 0.015), z = 437.09, *p* < 0.001. This indicates that at the reference (centered) levels of the smooth variables, the projected monthly incidence was exp(6.676) = 793 occurrences. The intercept for severe cases was 1.196 (SE = 0.139), z = 8.60, *p* < 0.001, indicating that at the reference levels, there were approximately 3.31 severe cases each month. The intercept for Deaths was − 2.658 (SE = 0.421), z = − 6.32, *p* < 0.001, indicating approximately 0.07 deaths per month at the reference levels. These critical intercepts establish the baseline mean counts; with the smooth terms (temperature, rainfall, trend, and seasonality) exhibiting genuine variance. Model calibration was further assessed using Pearson dispersion statistics (Table 4). Table 4 indicates that the Pearson dispersion diagnostics (the sum of Pearson residuals squared divided by residual degrees of freedom) demonstrated that the incidence GAM was well-calibrated (ϕ = 0.951). This aligns with the negative-binomial variance, which accounts for the observed variability and reinforces the dependability of standard errors and tests for this model. The severe-cases model demonstrated mild residual over-dispersion (ϕ = 1.319), suggesting residual heterogeneity or dependence not entirely captured by the smooth terms, which could be mitigated by integrating structure. The mortality model exhibited slight under-dispersion (ϕ = 0.760), a phenomenon frequently observed with sparse counts characterized by numerous zeros, where the negative binomial mean–variance relationship stabilizes residual variability. This conclusion aligns with our distinct zero-inflated model for mortality and reinforces the necessity for meticulous and robust uncertainty reporting. The dispersion profile endorses the choice of the negative-binomial family, with the severe model suggesting a possibility for minor improvement.

**Table 3:** Intercept estimates for NB-GAMs

model	Param	Estimate	Std.Error	z.value	*p*-value
Incidence	(Intercept)	6.676373	0.01527464	437.088590	< 1e-6
Severe	(Intercept)	1.196423	0.13919106	8.595544	< 1e-6
Deaths	(Intercept)	−2.658162	0.42065806	−6.319056	< 1e-6

^*^Significant level at 0.05

**Table 4:** Pearson dispersion statistics

Model	Pearson_Dispersion
Incidence	0.9507979
Severe	1.3189206
Deaths	0.7599551

### Model results and diagnostics

The generalized additive models (GAMs) revealed distinct dynamics for malaria incidence, severe cases, and mortality among children under five in Tarkwa-Nsuaem (2013–2023). For incidence, the baseline negative-binomial (NB) GAM identified a highly significant nonlinear long-term trend and seasonal cycle, while contemporaneous temperature and rainfall were not significant (Table 2). Figure S1 shows that incidence rose from ~ 500 monthly cases at the start of the record to over 1,000 in the mid-period, before declining after 2021. Variability scaled with incidence, supporting the NB assumption. Model comparisons (Table 5) indicated that incorporating lagged and accumulated rainfall substantially improved model fit (AIC = 1,627.3 vs. 1,707.0 for the baseline), while maintaining nearly identical explanatory power (Adj. R^2^ ≈ 0.80, deviance explained ≈ 0.80). By contrast, including AR(1) errors worsened performance (AIC = 1,724.8), consistent with the weak residual autocorrelation observed (Figure S4). These results confirm that incidence responds to recent precipitation history rather than contemporaneous totals, and that temporal autocorrelation is adequately captured by smooth trend and seasonality. Forecasts from the selected model indicate a modest decline with seasonal fluctuations, but incidence is expected to remain within the mid-hundreds to low-thousands range, requiring continued programmatic capacity (Table 7).

**Table 5:** Comparison of incidence model specifications

Model	AIC	Adj. R^2^ (mgcv)	Deviance explained
Incidence NB (baseline)	1,706.99	0.800	0.804
Incidence NB + lags/accum	1,627.29	0.797	0.800
Incidence GAM (bam) + AR(1)	1,724.84	0.738	0.747

Severe malaria, counts were initially high (> 150/month) but declined steeply through 2014–2016, stabilizing near zero thereafter (Figure S2). Only the smooth long-term trend was consistently significant (Table 2), while rainfall, temperature, and month terms were negligible (Figures S6, S8). The NB model captured variance scaling with the mean and provided a stable fit, though mild over-dispersion (ϕ = 1.32, Table 5) reflects residual heterogeneity. These results suggest that secular improvements in case management, rather than meteorological variation, drive the observed decline.

For mortality, deaths were rare, with sporadic 1–4 events in early years and a structural-zero regime after 2016 (Figure S3). The NB baseline model captured the secular decline (Table 2), but diagnostics indicated under-dispersion (ϕ = 0.76, Table 5) and excess zeros (Figure S7). A zero-inflated NB (ZINB) GAM substantially improved fit (AIC = 104.1 vs. 113.3, Table 6), consistent with the structural-zero regime. Partial-effect plots (Figure S5) confirmed that temperature had no explanatory power, rainfall exerted only a weak, near-linear effect, and the trend was nearly linear and negative. Together, these findings show that mortality dynamics are dominated by a long-term decline rather than short-term climate, and that dual-component models are more epidemiologically realistic at very low death counts.

**Table 6:** Comparison of baseline negative-binomial (NB) and zero-inflated negative-binomial (ZINB) generalized additive models (GAMs) for malaria deaths

Model	AIC	Adj. R^2^ (mgcv)	Deviance explained
Deaths NB (baseline)	113.25	0.152	0.411
Deaths ZINB GAM (ziplss)	104.08		

Parametric intercepts (Table 3) established baseline monthly outcomes at centered smooth values: ~ 793 cases for incidence, ~ 3.3 severe cases, and ~ 0.07 deaths. Pearson dispersion statistics (Table 4) confirmed good calibration for incidence (ϕ = 0.95), mild over-dispersion for severe cases, and under-dispersion for deaths. Collectively, these results support the use of NB GAMs with lagged rainfall terms for incidence, NB models for severe cases, and ZINB extensions for mortality.

### Forecasts of malaria incidence and mortality

**Fig. 3:**
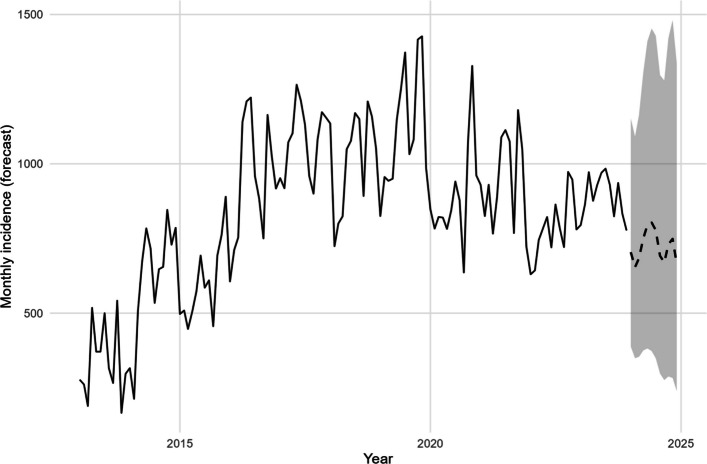
Forecasted monthly malaria incidence (2024)

**Fig. 4:**
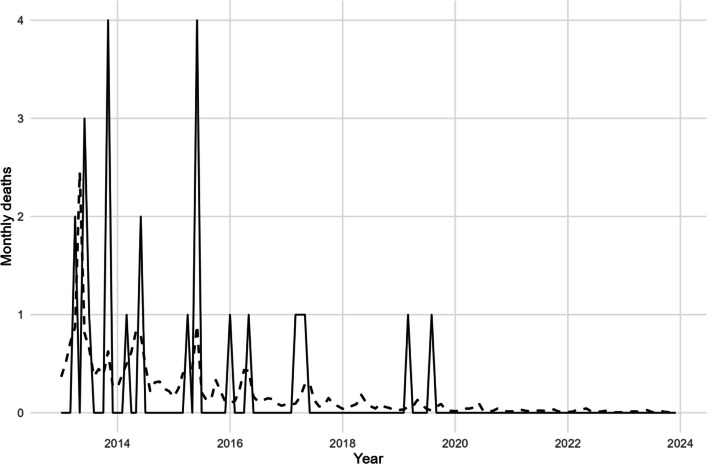
Forecasted monthly malaria deaths (2024)

Forecasts were generated using the best-performing models for incidence and mortality. For incidence, the rainfall-lagged NB-GAM projected a modest decline over the next 12 months, from ~ 667 cases/month in January 2024 to ~ 600 cases/month by December 2024 (Fig. 3; Table 7). Despite the gradual downward trajectory, predictive intervals were broad, ranging from ~ 250 to 1,350 cases. This reflects both the over-dispersed nature of case counts and the influence of rainfall memory on transmission. Model performance diagnostics confirmed robustness, explaining ~ 80% of observed variation (Adj. R^2^ = 0.797; deviance explained = 0.800) with minimal residual deviance (~ 0.85 per observation) (Table 8). These metrics indicate that the model reliably captures the main dynamics, even though substantial uncertainty surrounds extreme outcomes. For mortality, the zero-inflated NB-GAM provided a better fit than a standard NB model, consistent with the preponderance of structural zeros since ~ 2017. The model forecast a median of zero deaths per month in 2024, with 95% predictive intervals typically between 0 and 1, and occasional excursions up to 2 deaths during wetter months (Fig. 4). This forecast indicates stability at very low mortality levels, with limited month-to-month variability and no discernible seasonal or long-term trend.

**Table 7:** Forecast of monthly malaria incidence (2024)

Date	Forecast (mean)	PI 95% Lower	PI 95% Upper
2024–01-01	666.6	383	1,096
2024–02-01	669.4	372	1,138
2024–03-01	661.3	358	1,154
2024–04-01	653.4	343	1,174
2024–05-01	646.6	330	1,196
2024–06-01	639.7	316	1,220
2024–07-01	633.1	303	1,245
2024–08-01	626.3	290	1,271
2024–09-01	619.6	278	1,299
2024–10-01	613.2	266	1,327
2024–11-01	606.7	255	1,356
2024–12-01	600.4	244	1,386

**Table 8:** Performance diagnostics of the best NB-GAM

Model	N	Adj. R^2^ (mgcv)	Deviance explained	Deviance (total)	Deviance/N
Incidence NB + lags/accum	132	0.797	0.8	112.14	0.85

Together, these forecasts suggest that while malaria incidence is expected to persist at moderate levels with strong seasonal and rainfall-driven fluctuations, severe outcomes in the form of mortality are projected to remain rare and sporadic.

### Extended modeling, predictive validation, and forecasts incidence

#### Mortality: rare-event behavior and zero-augmentation (Fig. 6; Table 12)

Deaths remain rare with structural zeros in later years. In the split sample, a single-component NB slightly outperformed ZINB by AIC (115.5 vs 117.8; Table 12), but both frameworks confirm that trend is the only consistently informative smooth, with weather non-significant. Figure 6 shows realistic calibration at very low counts; for risk communication, interval-based forecasts are preferred over point accuracy.

### Twelve-month outlook and programmatic interpretation (Fig. 7)

Extending the validated incidence model produces a 12-month forecast characterized by seasonal undulation and wide, NB-consistent predictive intervals (mid-hundreds to low-thousands; Fig. 7). For mortality, forecasts concentrate at 0–1 events with occasional excursions during wetter periods. These outputs support advance positioning of RDTs/ACTs and staff around windows flagged by rainfall-memory indicators rather than calendar month alone.

### Incidence dynamics and rainfall “memory”

Adding rainfall memory to the baseline incidence NB-GAM (evident in Tables 9 and 10) improved information criteria and calibration (AIC dropped from 1269.3 to 1234.4; adjusted R^2^ and deviance explained ≈ 0.88), indicating that transmission tracks recent hydrometeorological history more than same-month totals. After pruning, fit remained strong (AIC ≈ 1248) the improvement is not an artifact of over-parameterization but comes from a parsimonious subset of memory and time terms. In the pruned model, trend (χ^2^≈334, *p* < 0.001) dominates the medium-term trajectory, while Rain_lag1 and Rain_roll6 are significant (p≈0.002), and temperature is monotonic within a narrow band (edf≈1). Apparent “calendar seasonality” becomes non-significant once rainfall memory and trend are included, implying that seasonal peaks are largely mechanistically explained by antecedent rainfall plus slow structural change.

**Table 9:** Incidence Model Comparison (Train)

Model	AIC	Adj. R^2^ (mgcv)	Deviance explained	Pearson dispersion
Incidence NB (baseline)	1,269.276	0.7610541	0.7779477	0.02843588
Incidence NB + lags/accum	1,234.423	0.8752080	0.8785908	0.01764071
Incidence NB (pruned)	1,247.966	0.8307796	0.8315390	0.02238268

**Table 10:** Significance of Smooth Terms (after pruning): Incidence (pruned)

Term	edf	Ref.df	Chi.sq	*p* -value
s(Temperature_imp)	1.00	1.00	9.47	0.002
s(Rain_lag1)	2.50	3.13	15.44	0.002
s(Rain_lag2)	1.00	1.00	2.13	0.144
s(Rain_roll6)	1.00	1.00	9.73	0.002
s(trend_mo)	7.36	8.35	334.12	<.001

### Observed vs. predicted (2013–2023) and out-of-sample realism

**Fig. 5:**
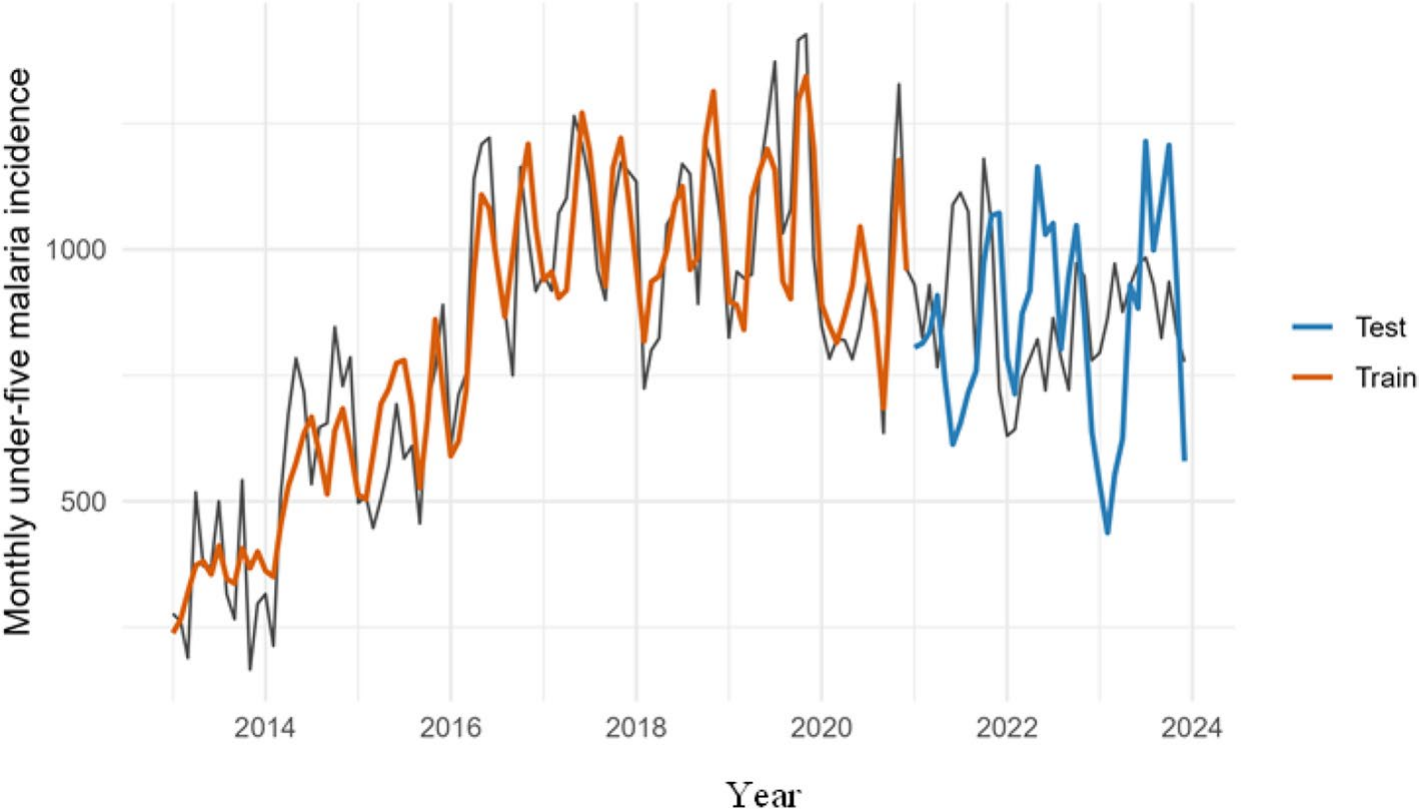
Model fit and out-of-sample predictions for monthly under-five malaria incidence (2013–2023)

Observed–predicted overlays confirm coherent reproduction of seasonal timing and peak amplitudes for incidence in both train and test periods (Fig. 5). Error profiles indicate limited degradation out of sample (MAE 96 vs 190; RMSE 116 vs 232 for train vs test, respectively; Table 11), supporting the model’s operational utility for preparedness (commodities and staffing). This pattern is consistent with rainfall-driven surges that the memory specification anticipates even when crest heights vary.

**Table 11:** Incidence: Predictive Performance (Train/Test)

Split	MAE	RMSE
Train (2013–2020)	96.21465	115.6732
Test (2021–2023)	190.01390	232.2216

### Severe cases: secular decline with minimal weather sensitivity

**Fig. 6:**
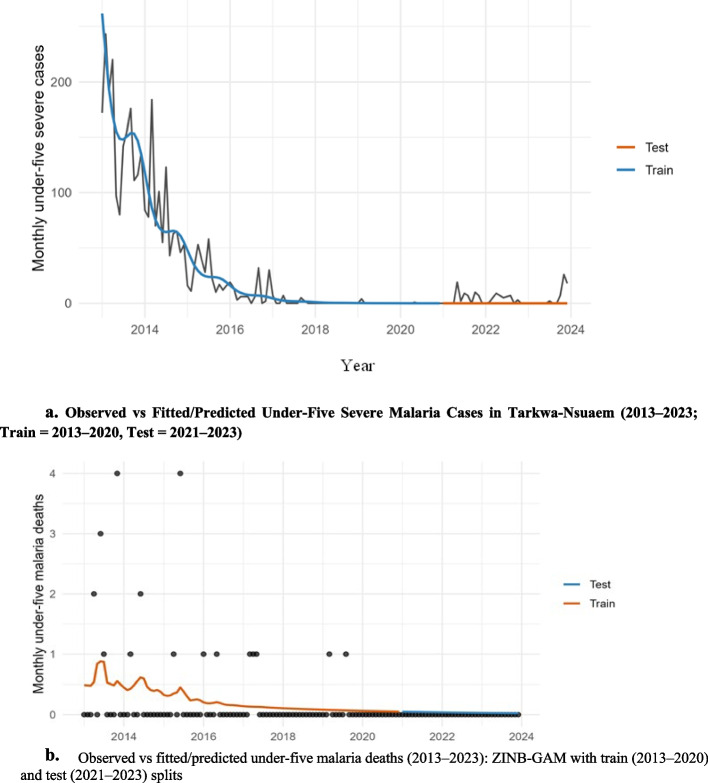
**a** Observed vs Fitted/Predicted Under-Five Severe Malaria Cases in Tarkwa-Nsuaem (2013–2023; Train = 2013–2020, Test = 2021–2023).** b** Observed vs fitted/predicted under-five malaria deaths (2013–2023): ZINB-GAM with train (2013–2020) and test (2021–2023) splits

After model pruning, only the long-term trend smooth was retained for severe malaria, yielding strong fit (≈ 85% deviance explained) while rainfall, temperature, and cyclic seasonality remained non-significant (see Table 9, Severe panel). The fitted trajectory reproduces the marked collapse from early high counts to near-zero by 2021–2023 with minimal residual structure, indicating that strengthened case management (rapid diagnosis, prompt ACT, streamlined referral) increasingly intercepts infections before progression to severe disease. This pattern implies a growing decoupling of severity from transmission intensity, consistent with programmatic gains rather than meteorological forcing (see Fig. 6, observed vs. fitted severe cases, 2013–2023).

### Mortality: rare-event behavior and zero-augmentation

Deaths remain rare with structural zeros in later years. In the split sample, a single-component NB slightly outperformed ZINB by AIC (115.5 vs 117.8; Table 12), but both frameworks confirm that trend is the only consistently informative smooth, with weather non-significant. Figure 6 shows realistic calibration at very low counts; for risk communication, interval-based forecasts are preferred over point accuracy.

**Table 12:** Deaths Model Comparison (Train)

Model	AIC
Deaths NB (baseline)	115.5200
Deaths ZINB (ziplss)	117.7888

### Twelve-month outlook and programmatic interpretation

**Fig. 7:**
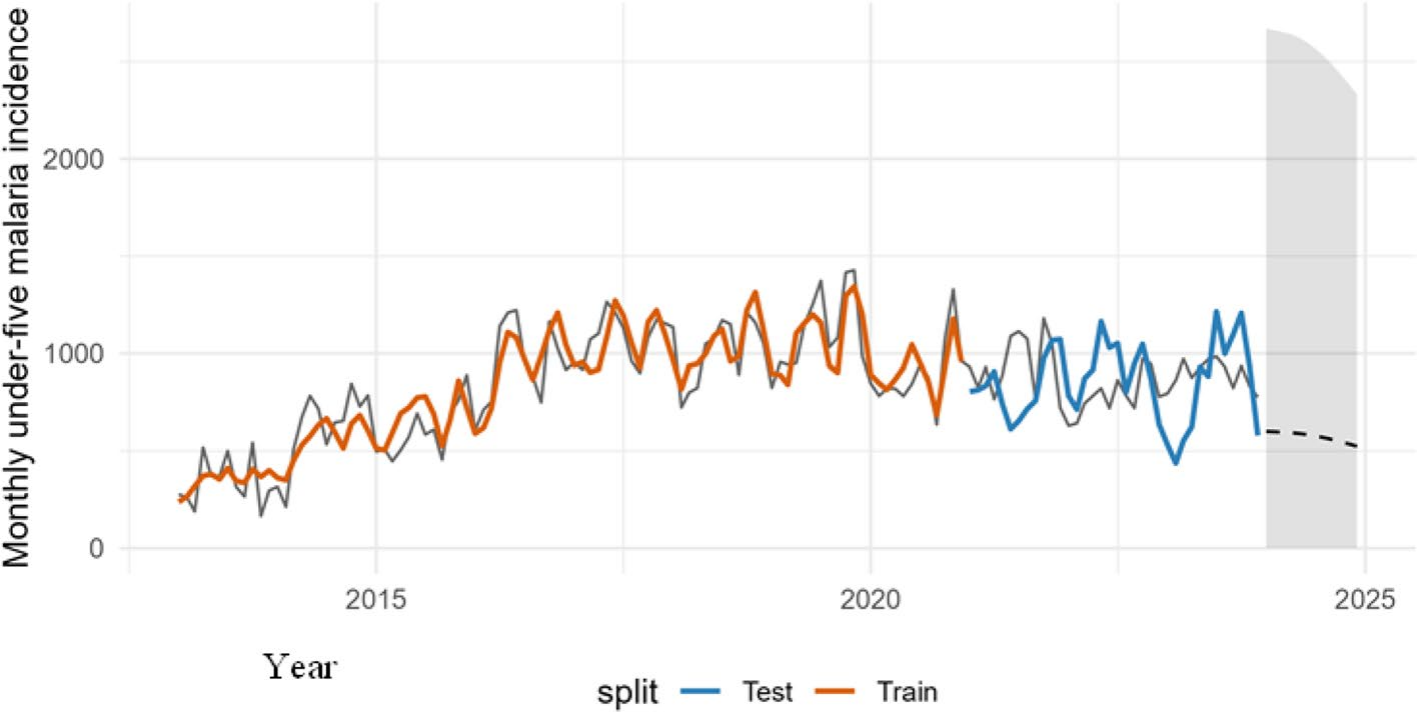
Observed (2013–2023) under-five malaria incidence with fitted/predicted values (train/test) and 12-month forecast (dashed) with 95% prediction interval (shaded)

Extending the validated incidence model produces a 12-month forecast characterized by seasonal undulation and wide, NB-consistent predictive intervals (mid-hundreds to low-thousands; Fig. 7). For mortality, forecasts concentrate at 0–1 events with occasional excursions during wetter periods. These outputs support advance positioning of RDTs/ACTs and staff around windows flagged by rainfall-memory indicators rather than calendar month alone.

The extended analyses clarify that short-lag and cumulative rainfall are the proximate climatic drivers of incidence in this setting, while explicit seasonality becomes redundant when rainfall memory and trend are included. Out-of-sample performance demonstrates that the model generalizes to unseen years with modest error growth, which is the appropriate standard for operational early warning. By contrast, severe disease and mortality remain predominantly governed by secular improvements in care pathways, with limited climate sensitivity at monthly resolution. These distinctions reconcile high, seasonal incidence with low downstream severity and deaths—an epidemiologically plausible pattern in systems with strengthened case management.

## Discussion

This study demonstrates that flexible, distribution-aware time-series models such as negative-binomial generalized additive models (NB-GAMs) are effective for monitoring malaria among children under five. Three principal findings emerged. First, incidence dynamics were dominated by a stable long-term trend and a modest seasonal cycle, rather than same-month meteorological conditions. In the baseline NB-GAM, trend and month-of-year terms were highly significant, while temperature and rainfall were not. Substituting same-month rainfall with lagged and accumulated indices (1–3 month lags, 3–6 month accumulations) improved likelihood fit (ΔAIC ≈ − 80) without changing explained variance. This indicates that malaria transmission is shaped by recent rainfall history rather than contemporaneous totals, consistent with entomological evidence that Anopheles mosquitoes reproduce and transmit disease weeks to months after heavy rainfall [21, 26, 33]. Similar patterns were observed by Abdulzeid et al. [1], in Tarkwa-Nsuaem, where rainfall significantly predicted incidence while temperature effects were negligible, though their SARIMAX framework did not explicitly model lagged or accumulated rainfall.

Second, severe cases and deaths declined sharply over the study period and were explained primarily by secular trends, not seasonality or climate. Severe malaria dropped dramatically between 2013 and 2016 and remained near zero thereafter, while mortality was rare and increasingly characterized by structural zeros. A zero-inflated NB-GAM improved model fit for deaths (ΔAIC = − 9), validating the need for an “excess-zero” framework that reflects intrinsically zero months punctuated by stochastic events. This finding aligns with Ghana’s declining under-five malaria mortality, attributed to widespread deployment of ACTs, ITNs, and improved case management [34]. Third, model diagnostics confirmed that NB likelihoods effectively handled over-dispersion (Pearson ϕ ≈ 1 for incidence), residual autocorrelation was negligible once rainfall memory and trend were modeled (making AR terms unnecessary), and out-of-sample forecasts were coherent. Projections suggest incidence will remain in the mid-hundreds to low-thousands, with wide prediction intervals reflecting real-world variability, while deaths are expected to remain near zero with occasional surges. These results carry important implications. Forecasting tools and early-warning dashboards should incorporate cumulative rainfall and lag indices rather than same-month totals to better anticipate short-term case loads. This would allow district programs to pre-position RDTs, ACTs, and staffing resources ahead of seasonal peaks. The divergence between persistent incidence and low mortality highlights progress in clinical management: despite ongoing transmission, the case-fatality burden has been reduced through improved access to diagnosis and treatment. For rare outcomes like deaths, prediction should emphasize event probabilities and count intervals, not mean-based errors, and zero-inflated or hurdle models are more appropriate.

District health managers should integrate rainfall-lag monitoring into early-warning dashboards to pre-position ACTs, RDTs, and staff before transmission peaks. Routine HMIS analyses can adopt NB-GAM templates to produce monthly alerts. Municipal vector-control units could synchronise LLIN and IRS campaigns with predicted rainfall accumulations. For mortality surveillance, incorporating zero-inflated models in national DHIMS analytics would improve accuracy where deaths are rare.

Several limitations should be acknowledged. First, the analysis relied on routine Health Management Information System (HMIS) data, which may be affected by reporting completeness, diagnostic stock-outs, or variation in clinical practices. Such factors could contribute noise to long-term incidence trends. Second, the study did not incorporate complementary meteorological (e.g., humidity), hydrological, or entomological indicators that influence malaria dynamics, nor did it include explicit time series of interventions such as LLIN distribution, IRS coverage, or ACT uptake. The absence of these variables limits attribution of the secular decline in severe cases and deaths to specific programmatic actions. Third, the forecasting framework employed a “hold-last” assumption for weather covariates, which may underestimate risks in years of extreme climate variability; incorporating seasonal climate forecasts could improve operational specificity. Fourth, the models were fitted to data from a single district, and while they demonstrate strong local performance, generalizability to other ecological zones in Ghana requires caution. Finally, while NB and zero-inflated NB GAMs captured over-dispersion and structural zeros effectively, alternative approaches such as distributed-lag nonlinear models, hierarchical Bayesian frameworks, or event-probability models may yield additional insights, especially for rare outcomes like mortality.

## Practical recommendations

District programs should track 1–3-month lags and 3–6-month rainfall accumulations as leading indicators in dashboards that trigger pre-positioning of RDTs and ACTs and surge staffing two to six weeks ahead of anticipated peaks. Vector-control micro-planning (drainage management, larval source reduction, and targeted LLIN/IRS outreach) should prioritize neighborhoods where rainfall-memory indicators signal durable aquatic habitats.

For mortality surveillance, planning should emphasize probability bands (0–1 events per month, with occasional excursions) and the timely minimization of delays to definitive care. Where zero inflation dominates, analytics and dashboards should adopt zero-augmented models to provide honest uncertainty around rare outcomes.

## Conclusion

This study advances malaria-surveillance analytics by demonstrating that rainfall-memory NB-GAMs can generate reliable, policy-useful forecasts from routine HMIS data. Beyond quantifying trends, the approach translates climate signals into actionable workload estimates, enabling timely logistics and staff planning. Future applications should couple this framework with intervention coverage data and regional climate forecasts to strengthen Ghana’s malaria early-warning and control systems. A negative-binomial generalized additive model with a smooth long-term trend and monthly cycle component effectively described almost 80% of the variance in incidence, whereas severity and mortality were mostly influenced by a secular decline rather than contemporaneous meteorological conditions. Substituting concurrent rainfall with short-lag (1–3 months) and accumulated (3–6 months) rainfall significantly enhanced the incidence model on the likelihood scale (ΔAIC ≈ − 79.7) without increasing model complexity, suggesting that transmission is influenced by recent rainfall history rather than the total of the current month. A zero-inflated negative binomial generalized additive model (NB GAM) surpassed a single-component negative binomial model in predicting deaths (AIC 104.1 vs 113.3), aligning with a structural zero framework and few occurrences. These findings operationally endorse an early-warning strategy that combines a consistent trend/seasonal baseline with rainfall-memory covariates to forecast workload many months in advance, acknowledging the broad predictive intervals characteristic of over-dispersed surveillance data. The incidence is anticipated to fluctuate between the mid-hundreds and low-thousands seasonally, while fatalities are projected to remain close to zero, with sporadic months exceeding this figure; thus, planning should ensure surge capacity for incidence while upholding effective clinical practices to minimize mortality. The negligible residual autocorrelation following smoothing suggests that incorporating an additional AR(1) error structure offers minimal benefit for standard forecasting. Future research should incorporate intervention timing and coverage, humidity and hydrology, and climate-forecast variables; examine parsimonious zero-augmented models for sparse outcomes; and investigate geographic expansions to address variability among facilities and communities. To this end, NB-GAMs—enhanced with lagged/accumulated rainfall and zero-inflation when applicable—provide an interpretable, policy-relevant framework for malaria surveillance: they effectively capture trends and seasonality, accommodate over-dispersion and excess zeros, and produce actionable forecasts for inventory, staffing, and targeted responses. These distinctions reconcile high, seasonal incidence with low downstream severity and deaths, an epidemiologically plausible pattern under strengthened case management. Embedding rainfall-memory NB-GAMs in district HMIS workflows can turn routine data into actionable, early warnings for stock, staffing, and targeted vector control.

## Supplementary Information

Supplementary Material 1.

## Data Availability

The secondary data on malaria incidence that supports the findings of this study are available from the authors with permission from the Western Regional Health Directorate through the District Health Information Management System (DHIMS) and are not publicly available.

## Abbreviations

ACF: Autocorrelation Function
ACT: Artemisinin-based Combination Therapy
AIC: Akaike Information Criterion
AR(1): First-order Autoregressive (error structure)
bam: *mgcv*’S “big additive models” fitting function
CFR: Case Fatality Ratio
CI/PI: Confidence Interval / Predictive Interval (here PI = forecast interval)
DHIMS-2: District Health Information Management System, version 2 (Ghana)
DHS/MIS: Demographic and Health Survey / Malaria Indicator Survey
edf: Effective Degrees of Freedom (for GAM smooths)
GAM: Generalized Additive Model
GLM: Generalized Linear Model
HMIS: Health Management Information System
IRS: Indoor Residual Spraying
ITN/LLIN: Insecticide-Treated Net / Long-Lasting Insecticidal Net
MAE/RMSE: Mean Absolute Error / Root Mean Squared Error
MAPE/sMAPE: Mean Absolute Percentage Error / Symmetric MAPE
NB: Negative Binomial (distribution/likelihood)
NB-GAM: Negative-Binomial Generalized Additive Model
R2 (Adj. R^2^): Coefficient of Determination (Adjusted)
REML: Restricted (Residual) Maximum Likelihood
RDT: Rapid Diagnostic Test (for malaria)
s(·): Smooth function term in a GAM
SMC: Seasonal Malaria Chemoprevention
U5: Under-five (children < 5 years)
WHO: World Health Organization
ZINB: Zero-Inflated Negative Binomial
ZINB-GAM (ziplss): Zero-Inflated NB GAM (fitted with *mgcv*’s ziplss family)
