# Supplementary material for: Malaria incidence, severity and mortality in children under five in Ghana: evidence from generalised additive models
Source: BMC Public Health. 2026 Jan 26;26:653. doi: 10.1186/s12889-025-25931-y (PMC12918577; doi:10.1186/s12889-025-25931-y)
Supplement: Supplementary file 1 — Supplementary Material 1. [file 12889_2025_25931_MOESM1_ESM.docx]

**Supplementary Material**


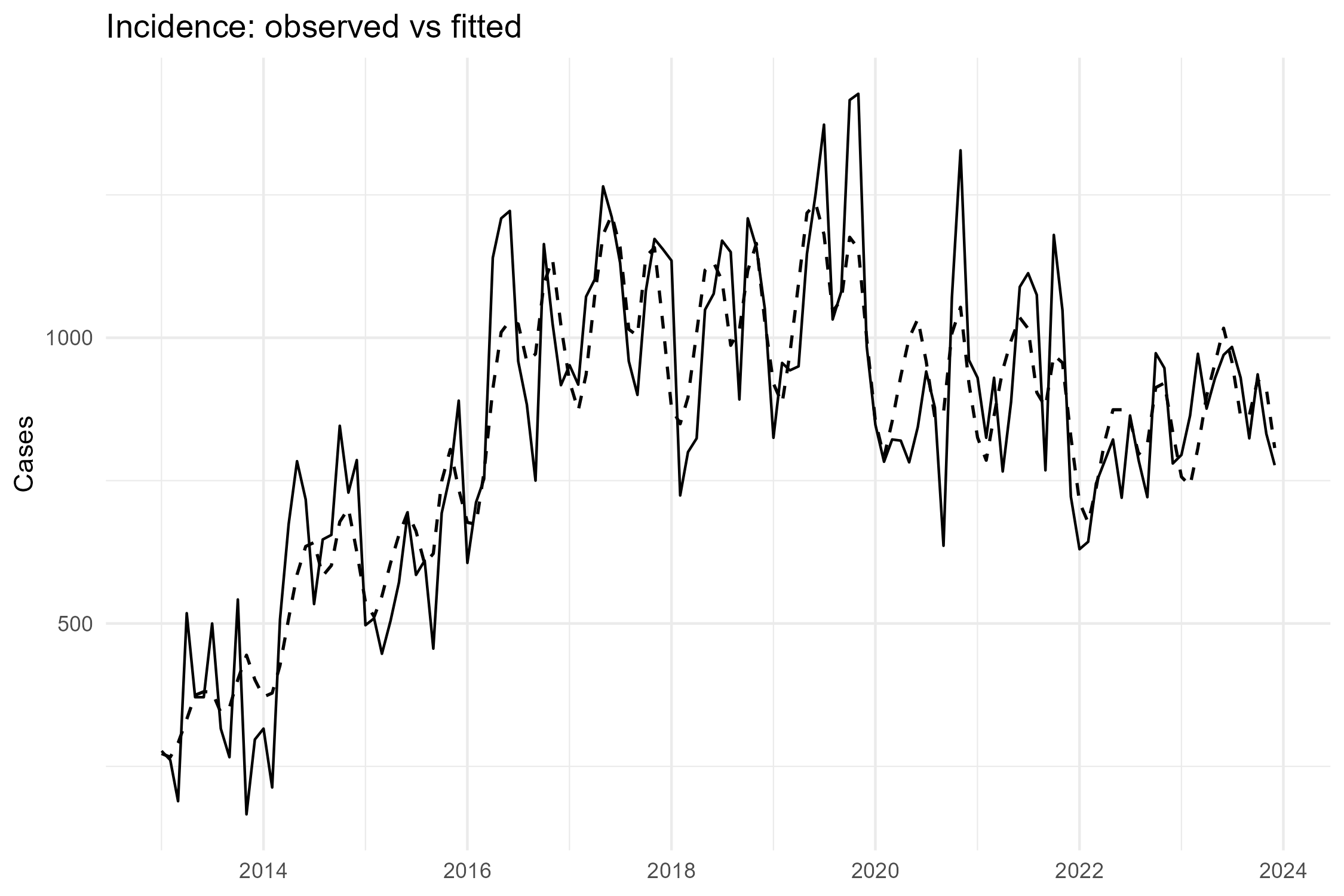


**Figure S1. Monthly malaria incidence with trend and seasonality, 2013–2024.**

**
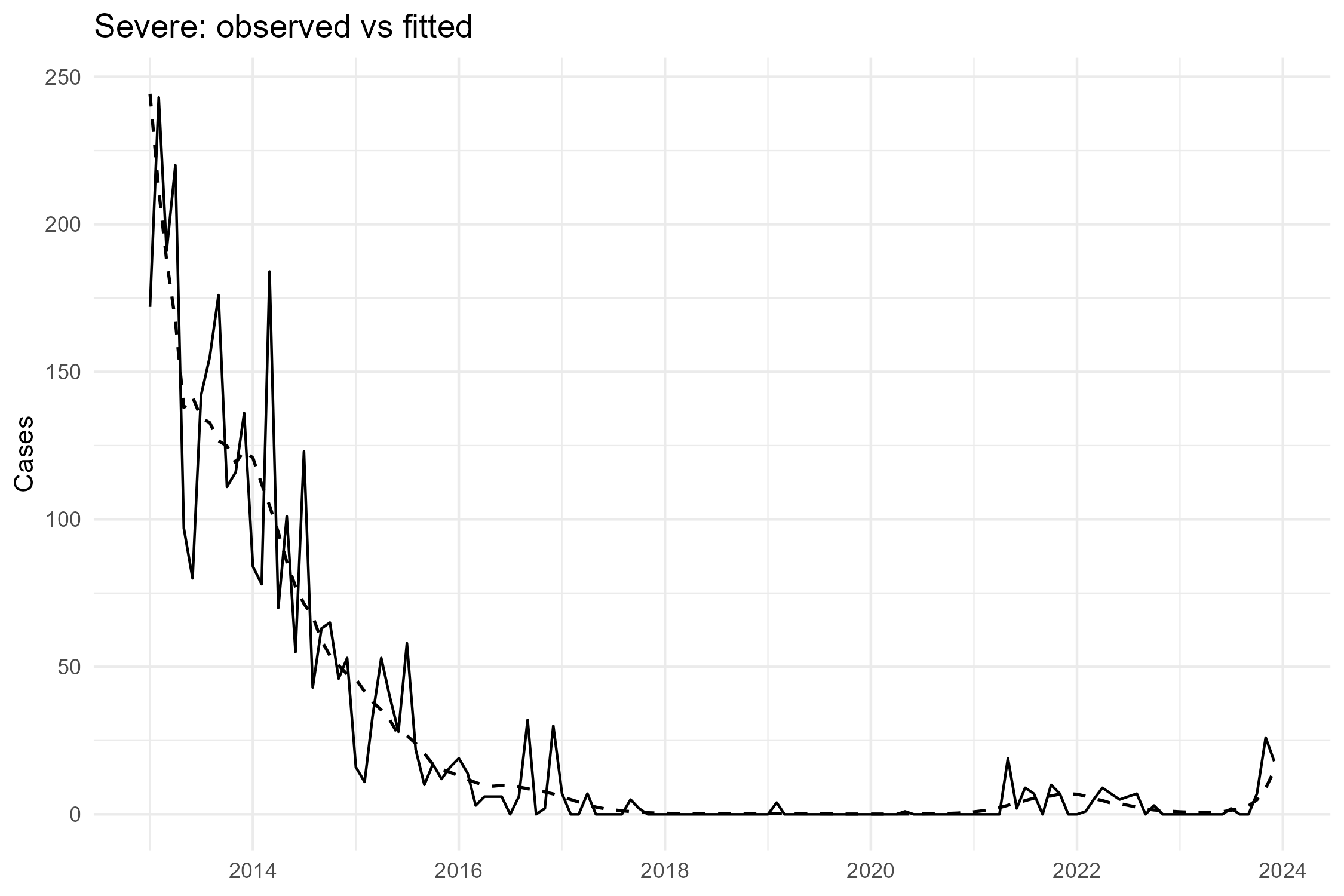
**

**Figure S2. Monthly severe malaria cases showing secular decline, 2013–2024.**


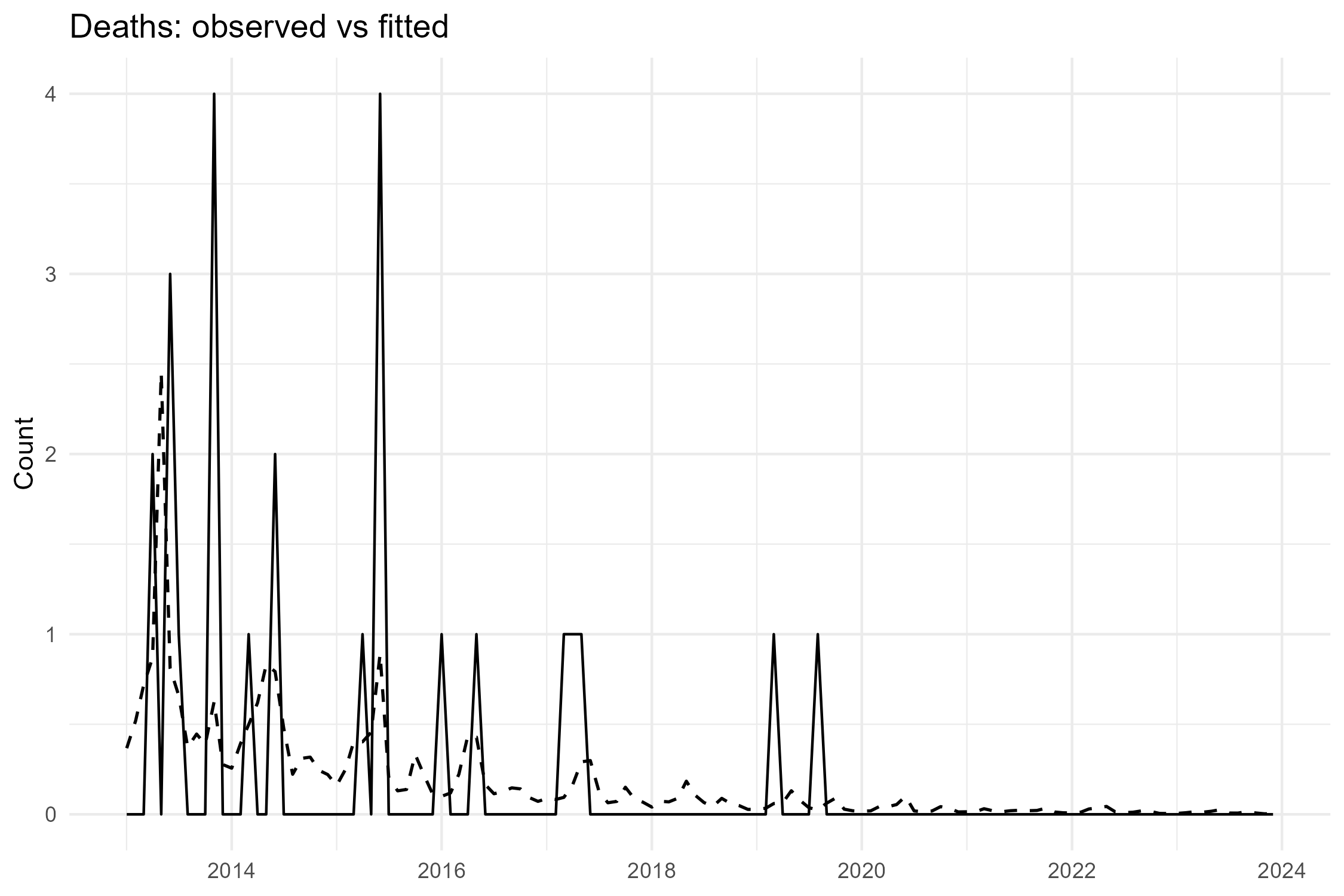


Figure S3. Monthly malaria deaths exhibiting a zero-inflated regime, 2013–2024


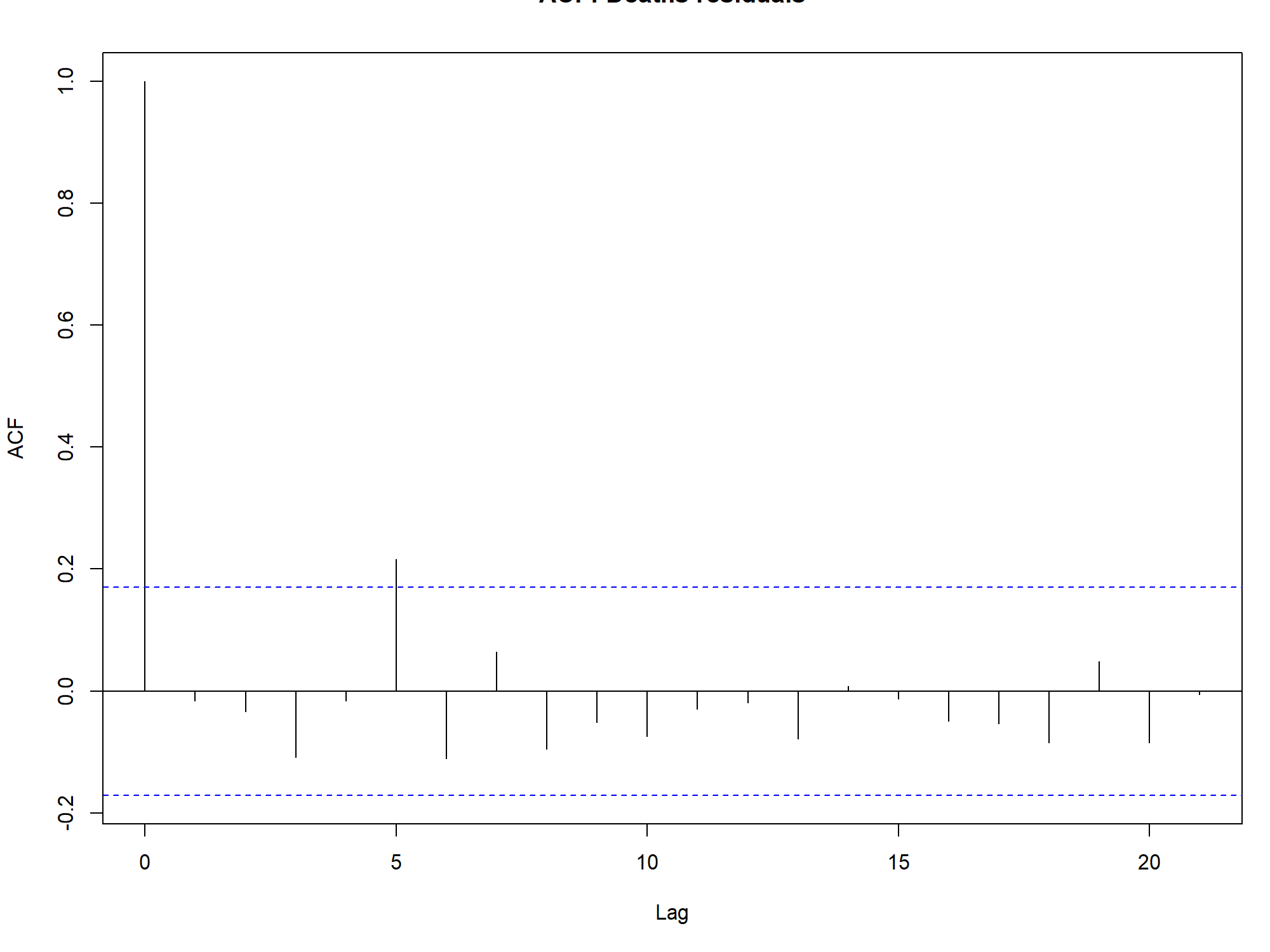


**Figure S4:** Autocorrelation of residuals for the malaria-deaths GAM (monthly data)


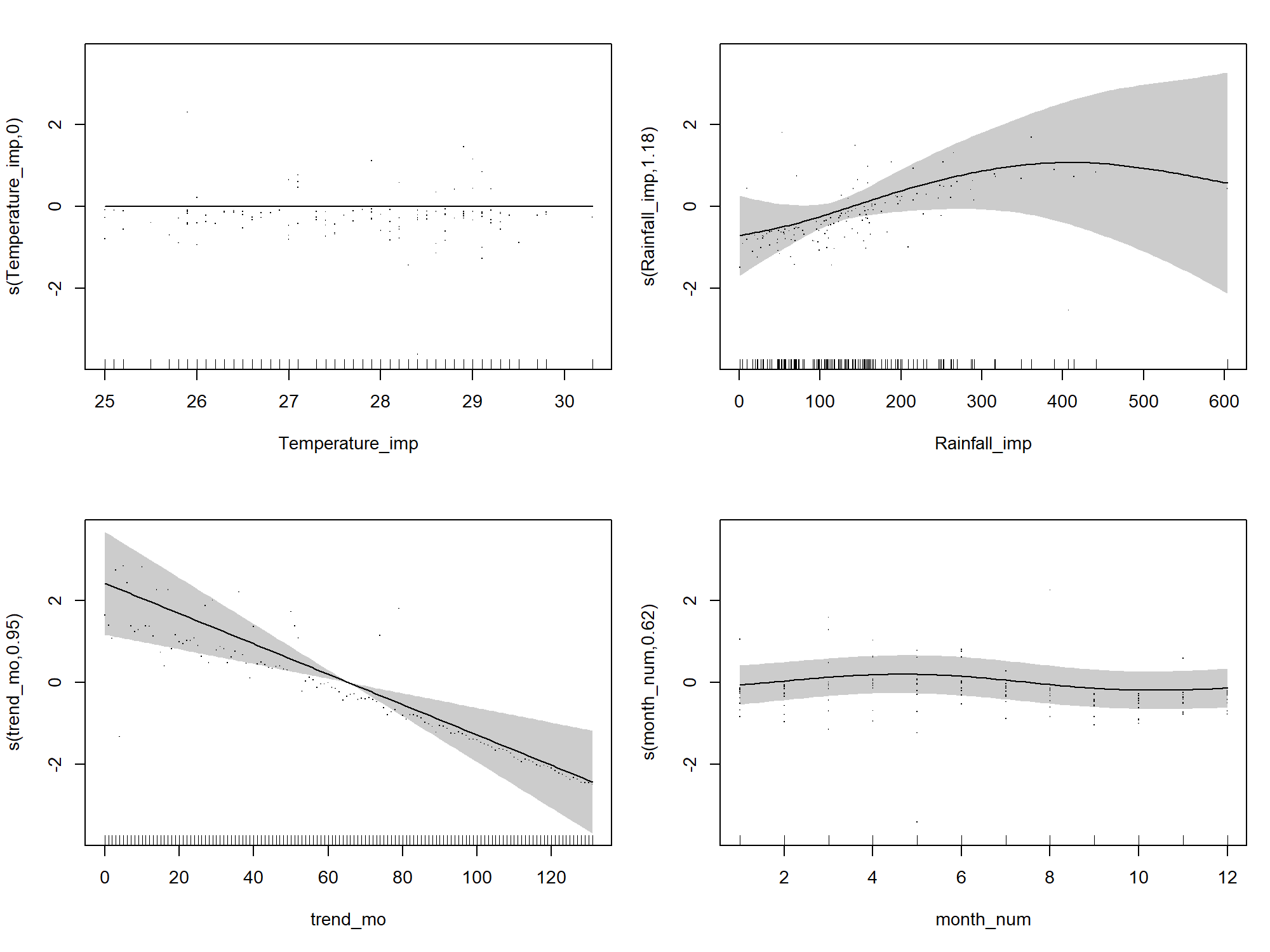


**Figure S5.** Partial smooth effects for the deaths model (Negative-Binomial GAM): temperature, rainfall, long-term trend, and cyclic month (solid line = estimated smooth on the log scale; grey band = 95% CI; rugs show data density).


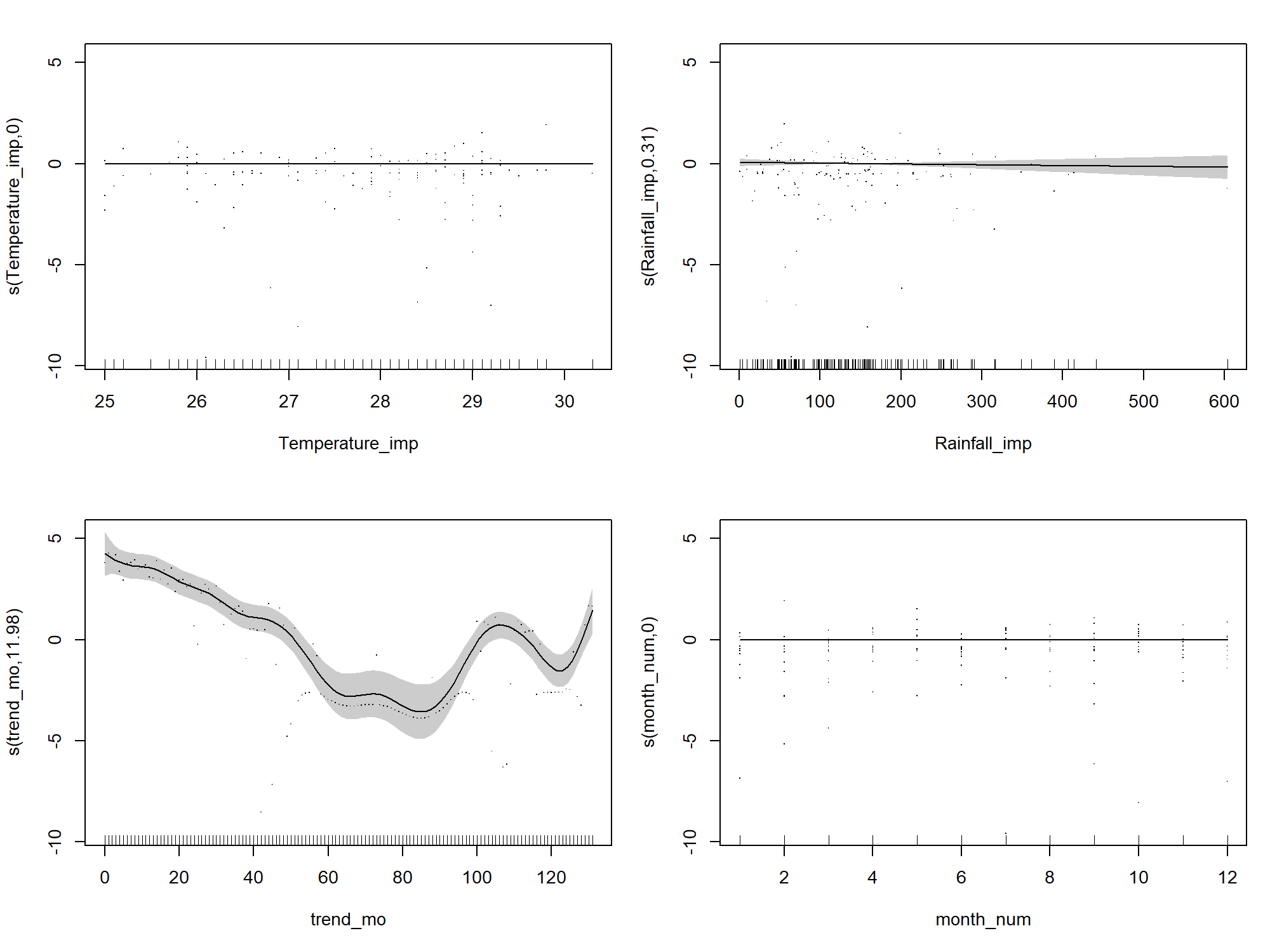


**Figure S6.** Partial smooth effects for the Severe‐case model (NB GAM): temperature, rainfall, long-term trend, and cyclic month (solid line = estimated smooth on the log scale; grey band = 95% CI; rugs show data density).


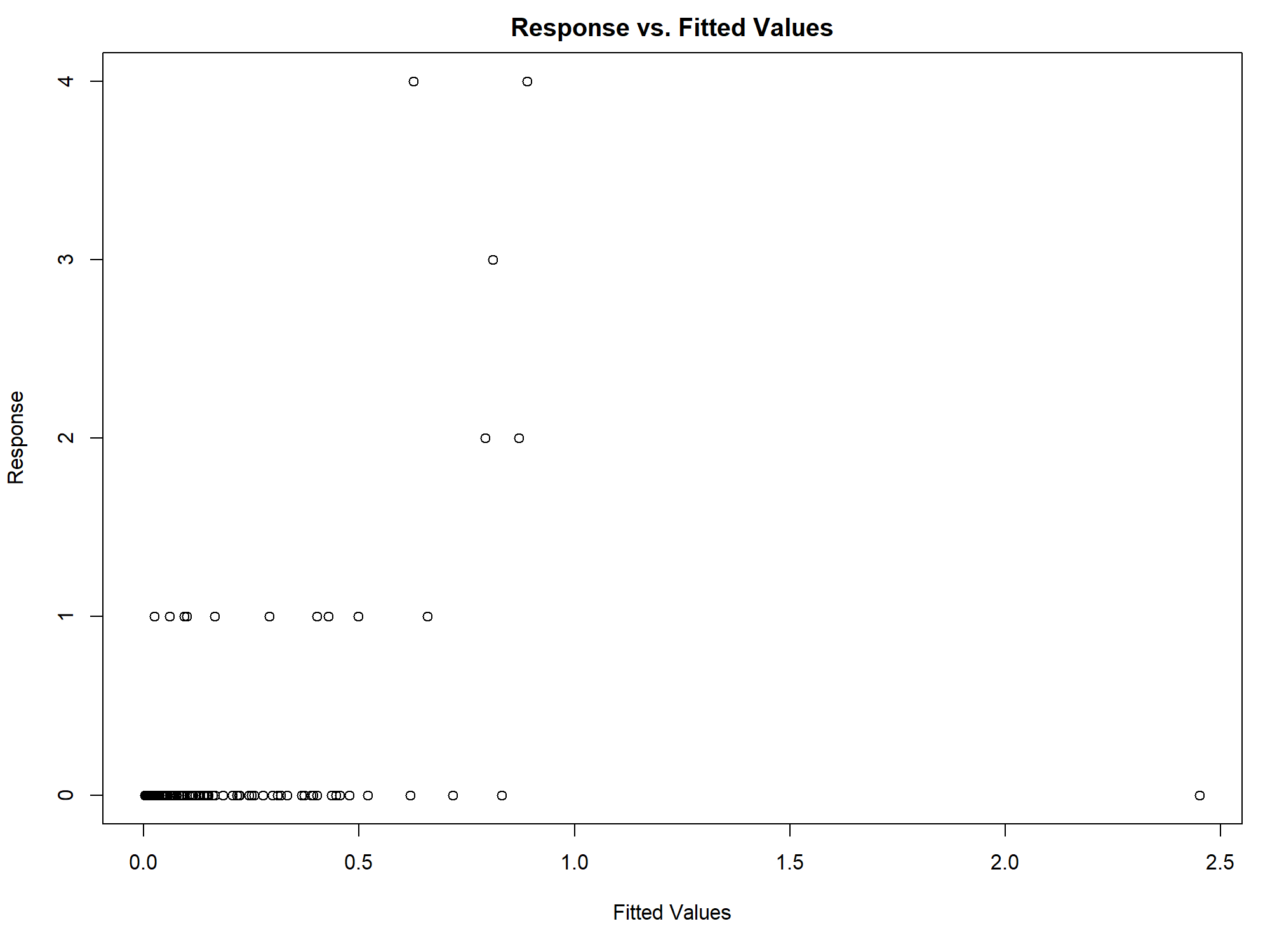


**Figure S7. Observed vs. Fitted Values for Monthly Under-Five Malaria Deaths (ZI-NB GAM)**

**
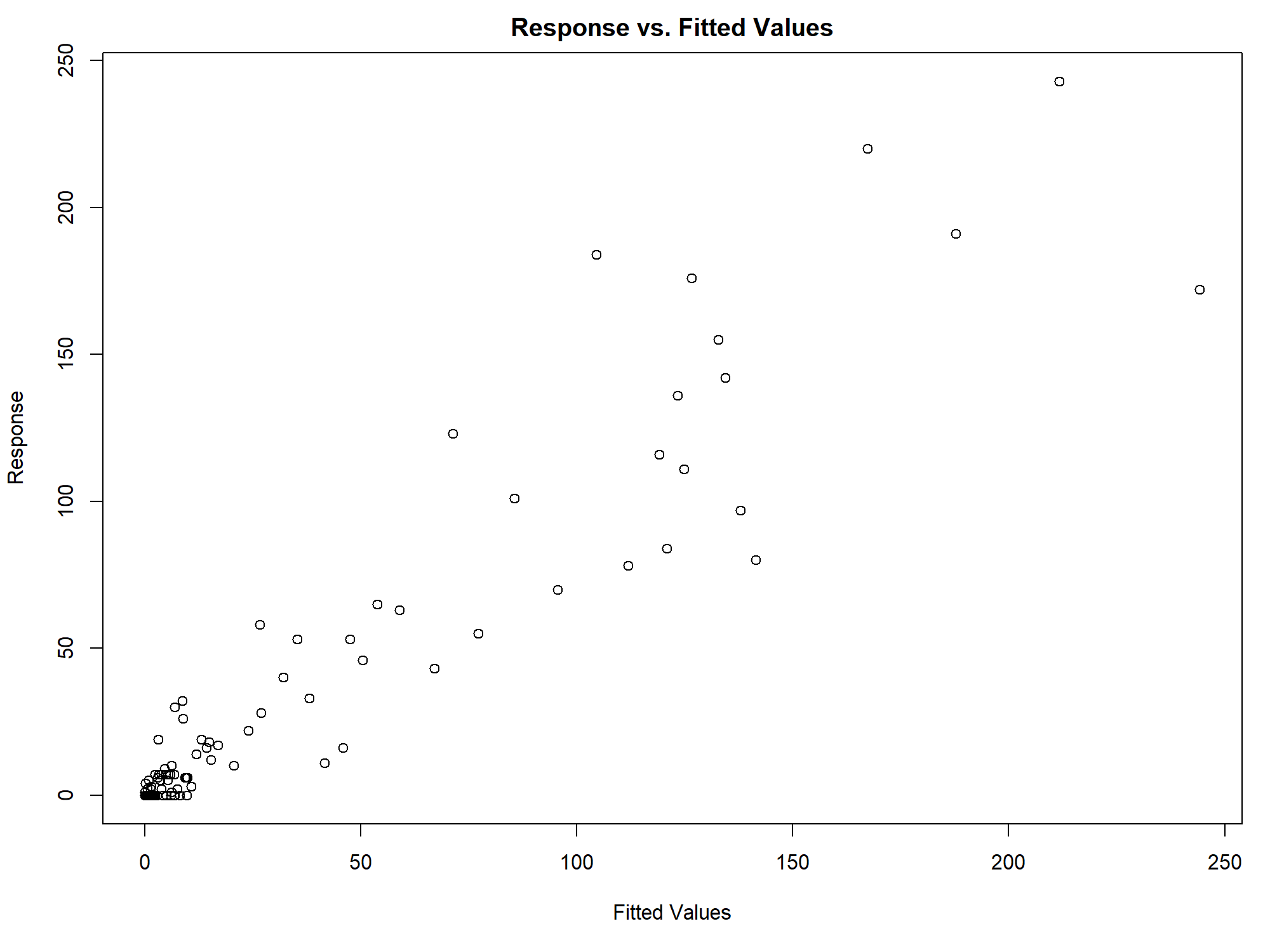
**

**Figure S8 — Observed vs. Fitted Values for Monthly Severe Malaria Cases (NB-GAM**
